# The complete mitochondrial genome of *Zelkova schneideriana* Hand-Mazz (Ulmaceae) and phylogenetic analysis

**DOI:** 10.1080/23802359.2021.1947913

**Published:** 2022-05-04

**Authors:** Xiao Liu, Hua-Bo Liu, Peng Yin, Ji-Chen Xu

**Affiliations:** National Engineering Laboratory for Tree Breeding, Beijing Forestry University, Beijing, China

**Keywords:** Mitochondrial genome, phylogenetic analysis, *Zelkova schneideriana* Hand-Mazz

## Abstract

*Zelkova schneideriana* Hand-Mazz is a second-class key protected wild plant in China. Here, the complete mitochondrial genome of *Zelkova schneideriana* Hand-Mazz was sequenced using Nanopore Sequel and Illumina NovaSeq platform. The mitochondrial genome was assembled into three circular-mapping molecules with the genome sizes of 154,640 bp, 192,388 bp and 146,907 bp, including 36 protein-coding genes, 19 tRNA genes, and 3 rRNA genes. Phylogenetic analysis indicated that *Zelkova schneideriana* Hand-Mazz is close with *Hemiptelea davidii*, a species in same *Ulmaceae Mirb*.

Zelkova is a genus of Ulmaceae with 6 species mostly distributed in southwest Europe, southwest Asia, and east Asia (Christe et al. 2014). As a deciduous tree species, Zelkova is even a second-class key protected plant in China. *Zelkova schneideriana* Hand-Mazz is the major species widely planted in China (Guo et al. 2014). The timbers of *Zelkova schneideriana* Hand-Mazz were dense, hard, beautiful grain and corrosion resistant suitable for manufacturing high valued furniture. Because of the leaves with seasonal variations in color, *Zelkova schneideriana* Hand-Mazz was also widely planted using as a landscape tree species in recent years (Luo et al. 2011). In this study, we first sequenced and assembled the complete mitochondrial genome of *Zelkova schneideriana* Hand-Mazz using high-throughput sequencing technique. Thus would help us well understand the status and characterization of *Zelkova schneideriana* Hand-Mazz in evolution.

The plant leaves were harvested from the nursery of Qingdao Boqing Garden Engineering Co., Ltd, Qingdao of Shandong Province, China (36.49°N, 120.57°E). The voucher specimens were conserved at Beijing Forestry University under the voucher number ZS-2020-001. The whole genomic DNA was isolated from fresh leaves using the NexteraXT DNA Library Preparation Kit (Illumina, San Diego, CA). The constructed library with an average length of 350 bp was then sequenced on the Illumina NovaSeq 6000 platform and Nanopore Sequencing platform, respectively. The clean reads were obtained by removing the contaminated and low-quality sequences. The mitochondrial genome was assembled by SPAdes v3.11.0 software (Antipov et al. 2016). Genome was annotated using UGENE v36.0 (Okonechnikov et al. 2012) and tRNAscan-SE v2.07 (Lowe and Eddy 1997).

The mitochondrial genome of *Zelkova schneideriana* Hand-Mazz was assembled into three circular-mapping with the genome sizes of 154,640 bp (mt1), 192,388 bp (mt2) and 146,907 bp (mt3). The GC content of mitochondrial is 44.6%, 45.2% and 45.2%, respectively. The mt1 genome contains 28 protein-coding genes and 6 tRNA genes, while mt2 contains 31 protein-coding genes and 9 tRNA genes, and mt3 contains 20 protein-coding genes, 4 tRNA genes and 3 rRNA genes. Totally the mitochondrial genome of *Zelkova schneideriana* Hand-Mazz contains 58 unique genes, including 36 protein-coding genes, 19 tRNA genes, and 3 rRNA genes. The annotated mitochondrial genome has been submitted to NCBI database under accession number BankIt2436389 mt1 MW717907, BankIt2436389 mt2 MW717908, and BankIt2436389 mt3 MW717909, respectively.

Seventeen common protein-coding genes (*atp1*, *atp4*, *atp8*, *atp9*, *cox1*, *cox2*, *cox3*, *matR*, *nad2*, *nad3*, *nad4*, *nad4L*, *nad5*, *nad6*, *nad7*, *nad9*, *rps3*) were aligned with the homologous genes in other 14 species using MAFFT v7.471 (Katoh et al. 2019). Phylogenetic analyses were performed using maximum likelihood (ML) with RAxML v8.2.12 (Stamatakis, 2014) based on GTRGAMMA model with 1000 bootstrap replicates. The phylogenetic tree showed that *Zelkova schneideriana* Hand-Mazz is close with *Hemiptelea davidii* (genetic distance 0.013312) (Figure 1), which is the only *Ulmaceae* species included (Liu et al. 2019).

**Figure 1.**
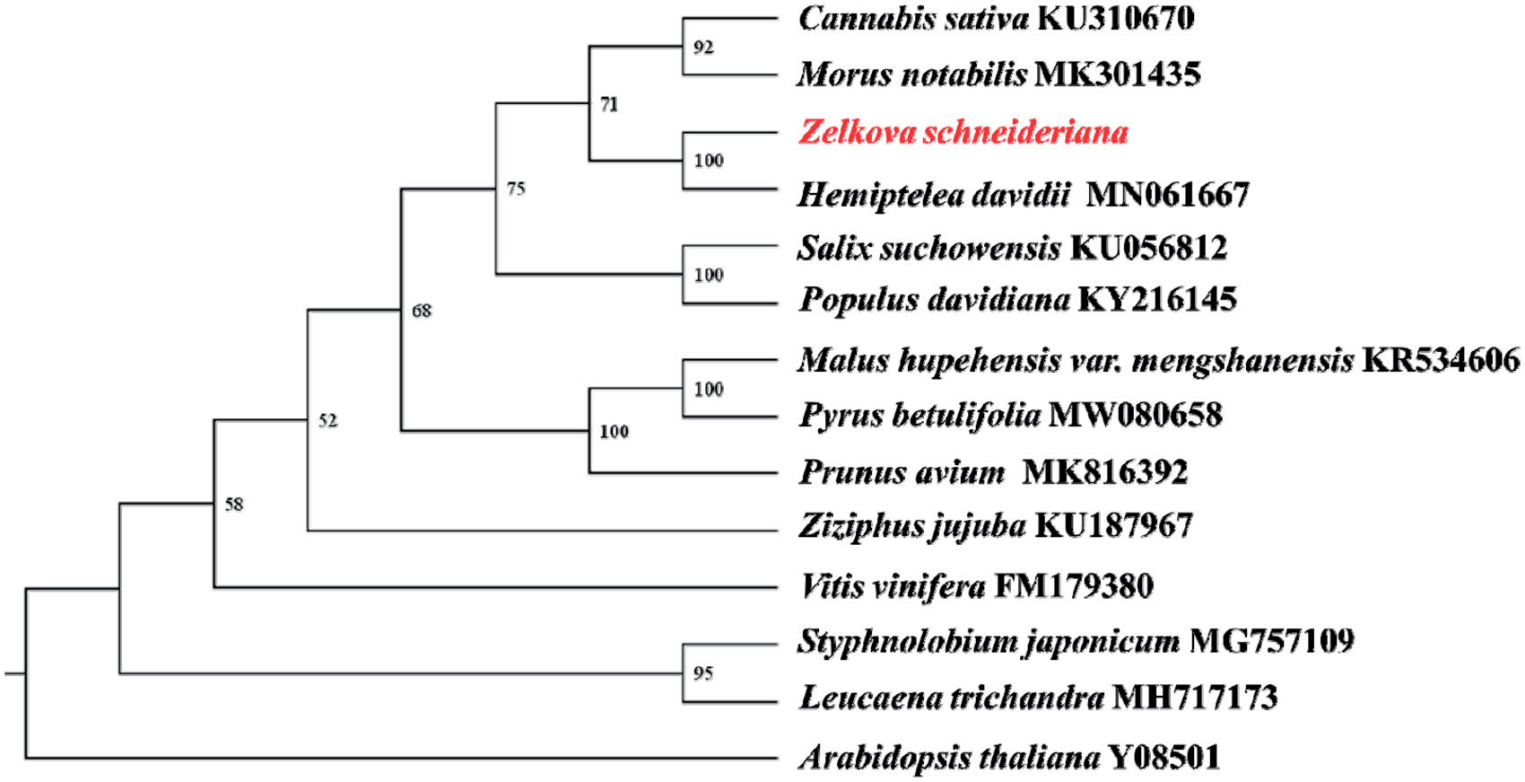
The phylogenetic tree of 14 plant mitochondrial genomes based on 17 common protein-coding genes using *Arabidopsis thaliana* as an out-group. The number on each node indicates the bootstrap value.

## Data Availability

The sequence data that support the findings of this study are openly available in GenBank of NCBI at (https://www.ncbi.nlm.nih.gov/) under the accession number BankIt2436389 mt1 MW717907, BankIt2436389 mt2 MW717908, and BankIt2436389 mt3 MW717909, respectively.
